# Highlights of ASCO 2024

**DOI:** 10.1093/oncolo/oyae250

**Published:** 2024-09-21

**Authors:** Alfred I Neugut, Susan E Bates

**Affiliations:** Department of Medicine, Herbert Irving Comprehensive Cancer Center, Columbia University, New York, NY, United States; Department of Epidemiology, Vagelos College of Physicians & Surgeons, Columbia University, New York, NY, United States; Mailman School of Public Health, Columbia University, New York, NY, USA; Department of Medicine, Herbert Irving Comprehensive Cancer Center, Columbia University, New York, NY, United States; Department of Epidemiology, Vagelos College of Physicians & Surgeons, Columbia University, New York, NY, United States; James J. Peters Bronx VA Medical Center, Bronx, NY, United States

## Abstract

Highlights from the ASCO annual meeting are presented.

Once again, this year, we present highlights from the annual meeting of the American Society of Clinical Oncology, held in Chicago, with over 40,000 attendees and over 5,000 oral and poster presentations. A large number of studies were interesting for a variety of reasons, so of course we had to be selective in what we could include and share below with the readership of *The Oncologist*. The abstracts can be accessed at ASCO.org. Where possible, we have also included the citation for the newly released concurrent publication—please forgive any omissions.

## Treating lung cancer

ASCO 2024, had several very interesting lung cancer studies.

- **Late-Breaking Abstract 4** (**LBA4**),^1^ presented in the Plenary Session, further expanded our understanding of the role of osimertinib in patients with non-small cell lung cancer (NSCLC) harboring epidermal growth factor receptor (EGFR) mutations. Osimertinib has previously been shown to be efficacious in prolonging survival for the 15% of patients with metastatic NSCLC whose tumors harbor EGFR mutations.^2,3^ The LAURA study randomized patients with unresectable stage III NSCLC with EGFR mutations who had been treated with definitive platinum-based *chemoradiotherapy* and had not experienced progression of disease to receive either osimertinib (*n* = 143) or placebo (*n* = 73) until disease progression.^4^ Anti-tumor activity, reflecting the impact of the prior chemoradiotherapy and the trial intervention (placebo or osimertinib), was notably higher with the addition of osimertinib, but the 2% complete response rate reminded us that this therapy is not curative. The median progression-free survival (PFS) was 39.1 months for the osimertinib group vs 5.6 months for placebo hazard ratio (HR) 0.16, 95% confidence interval (95%CI; 0.10-0.24). With data at only 20% maturity, overall survivals (OS) were similar and likely not to achieve significance given that 81% of patients randomized to placebo eventually received osimertinib after progression. While the authors concluded that this established a new standard of care for the use of osimertinib for patients in this setting, questions remain unanswered. Toxicity resulting from osimertinib was not trivial, with radiation pneumonitis (48% with osimertinib vs 38% with placebo), diarrhea (36% vs 14%), and rash (24% vs 14%) most common. Adverse events ≥grade 3 were reported in 50 patients (35%) with osimertinib and 9 patients (12%) with placebo. Interstitial lung disease was reported in 11 patients (8%) receiving osimertinib and 1 patient (1%) receiving placebo. While osimertinib is unarguably valuable, whether its start could be delayed a few months until recovery from chemoradiotherapy to possibly reduce complications needs to be addressed. If OS remains similar and especially if toxicity with osimertinib in the placebo group who received it later is recorded and found to be lower, one could envision a delay in its start of several months as a better alternative. Results from this study can be contrasted to the results of the ASCENT trial, in which afatinib was given as induction therapy for 2 months before it was combined with concurrent chemoradiotherapy in patients with locally advanced NSCLC, and continued for 2 years after chemoRT.^5^ As in the LAURA study, complete responses were rare; the median PFS was 2.63 years (95%CI, 1.41-3.07), with most patients experiencing disease progression after discontinuation of the tyrosine kinase inhibitor (TKI). Both studies remind us that much work remains to be done.
- While it seems not so long ago, it was 2006 when clinical trials with crizotinib began, and 2007 when the first patient with NSCLC harboring an ALK mutation was enrolled (about 5% of NSCLC harbor an ALK mutation).^6,7^ Eighteen years later, the results of the CROWN trial (Abstract LBA8503)^8^ comparing lorlatinib to crizotinib in 296 patients with untreated ALK-positive advanced NSCLC were presented. Lorlatinib a third-generation ALK inhibitor with a chemical structure different from other ALK TKIs, was designed to be active against most resistant mutations identified with first- and second-generation ALK inhibitors. At 60 and 55 months of follow-up, respectively, PFS at 5 years, the primary endpoint, was 60% and 8%, with lorlatinib and crizotinib, respectively, a HR for progression of 0.19 (95%CI, 0.13-0.27). Equally remarkable, lorlatinib was associated with marked efficacy against existing intracranial disease, while also reducing its development. After 5 years of follow-up, in patients with brain metastases at baseline the response to lorlatinib was very high, with the majority complete and durable responses, and intracranial progression in only 5/35 patients. Amongst patients who entered the study without brain metastases, 96% (110 of 114) of the lorlatinib cohort was free of intracranial disease compared to 64% (70 of 109) of those treated with crizotinib.^9^ Designed to avoid active transport by efflux transporters such as P-glycoprotein, and able to cross the blood–brain barrier because of its lipophilic nature, lorlatinib has excellent CNS penetration and the highest cerebrospinal fluid (CSF) to free plasma drug ratio of all clinical ALK inhibitors. Likely the most successful third-generation targeted therapy, with efficacy exceeding that of even osimertinib, it has taught us the value of rational drug development in targeted therapy that succeeds best when active first-generation agents guide the way.
- A third lung cancer study, ADRIATIC (Abstract LBA5),^10^ evaluated the value of durvalumab in patients with limited stage small cell lung cancer (SCLC) by randomly assigning patients whose disease had not progressed following concurrent chemoradiation and had completed standard of care treatment to receive either placebo plus placebo followed by placebo, durvalumab plus placebo followed by durvalumab, or durvalumab plus tremelimumab followed by durvalumab until progression or up to 24 months. With the durvalumab plus tremelimumab arm blinded until the final analysis, the presentation focused on the benefit with durvalumab (*n* = 264) vs placebo (*n* = 266) at the preplanned interim analysis, with a median follow-up of 37 months. The median PFS was 16.6 months for durvalumab vs 9.2 months for placebo (HR 0.76, 95%CI 0.61-0.95); the median overall survival was 55.9 months for durvalumab vs 33.4 months for placebo (HR 0.73, 95%CI 0.57-0.93). With similar but not identical rates of severe adverse effects, the investigators concluded that durvalumab should now be a standard of care for patients with limited stage SCLC after chemoradiation. With less than 10% of patients out 4 years, ADRIATIC has yet to reach the maturity required for confidence in the OS analysis, but will no doubt be successful with only the magnitude of benefit to be determined. In a disease where only 30%-35% of patients survive 5 years even with aggressive curative-intent radiation therapy, the emerging data suggests that the control arm will emulate that outcome, with durvalumab poised to possibly raise that to a value close to 50%. Interestingly, despite an impressive 22-month OS differential the HR is only 0.73, with curves nearly parallel slightly above and below the 50% threshold, suggesting that the difference is likely less than scored. Gratifyingly, the curves are trending to a wider separation over 5 years. Compared with the very modest gains achieved in extensive stage SCLC with atezolizumab, the more substantial advance emerging with durvalumab in ADRIATIC will be seen by many as evidence of greater immunotherapy efficacy earlier in the natural history of a disease, an argument confounded by the almost certain differences in the biology of extensive and limited stage disease.
- Granted accelerated approval by the Food and Drug Administration (FDA) 2 wk before ASCO 2024^11^ for use in extensive stage SCLC with disease progression on or after platinum-based chemotherapy, tarlatamab-dlle entered the meeting facing high expectations that it was unable to meet. The accelerated approval for this bispecific T-cell engager (BiTE) that binds DLL3 on cancer cells and CD3 on T cells was the first time that a BiTE had been approved for a solid tumor. Leveraging the data from the DeLLphi-301 trial^12^ that provided evidence of efficacy for the accelerated approval, **Abstract 8015**^13^ presented a subgroup analysis of the phase 2 study by the presence or absence of stable brain metastases at presentation. While previous data has shown activity against brain metastases with pembrolizumab, nivolumab, atezolizumab, durvalumab and cemiplimab as naked antibodies, uncertainty remains as to whether a BiTE dragging a T-cell might possess such activity. In 17 of 23 patients treated with brain metastases at baseline among the 100 patients who enrolled in DeLLphi-301, CNS tumor shrinkage ≥30% was observed in 10 patients and <30% in 7 patients. However, the authors acknowledged that prior radiation therapy confounded the attribution of this observation to tarlatamab-dlle, with a median time from radiotherapy to tumor size nadir of just 21 wk for the 17 patients, and only 3 being longer than 34 wk, at 50, 120, and 144 wk, respectively. Given that brain metastases can take days or weeks to start shrinking after radiation therapy, and months or even years for the tumor to reach its nadir, the intracranial efficacy of tarlatamab-dlle remains unknown.

## Treatment of oligometastatic colorectal cancer

Three trials in colorectal cancer reported on the management of oligometastatic disease in the liver.

- **Abstract LBA3501** ^14^ compared the outcomes of 148 patients randomly assigned to surgical resection with that of 147 treated with thermal ablation. After a median follow-up of 28.8 months, there was no difference with regard to OS (HR 1.042; 95%CI 0.689-1.576; *P* = .846). However, safety data favored ablation with fewer adverse events (*P* < .001), reduced hospital stay (4 days vs 1 day, *P* < .001), and reduced mortality (2.1% (*n* = 3) vs 0% (*n* = 0)). The authors concluded that, in patients with liver metastases≤3 cm in size, thermal ablation would reduce complications, shorten hospital stays and improve local control, without compromising disease-free and overall survival. Given that surgical data has shown that the resection of liver metastases in colorectal cancer can improve long-term outcomes for some patients and, with isolated liver metastases, can be potentially curative, this strategy could expand the number of patients able to undergo therapy of localized disease.
- But as the ORCHESTRA trial (**LBA 3502**)^15^ found, the extent of resections has a limit. With 192 patients randomized to continue chemotherapy alone and 190 to chemotherapy and surgical debulking consisting of resection of multiple metastases (>5 if liver-only) or multiorgan metastases, the more radical surgical strategy did not improve outcomes. The median PFS values were 10.5 and 10.4 months, in the experimental and standard arms, respectively (adjusted HR 0.83, 95%CI 0.67-1.02, *P* = .076), with median OS scoring 30.0 and 27.5 months (adjusted HR 0.88, 95%CI 0.70-1.10, *P* = .225).
- Finally, the TRANSMET study (Abstract 3500)^16^ assessed the efficacy of liver transplantation combined with chemotherapy vs chemotherapy alone in patients with unresectable BRAF wild-type colorectal liver metastases, with response to chemotherapy for >3 months, who had received <3 lines of chemotherapy, and were without extrahepatic disease. Between February 2016 and July 2021, 47 patients were randomized to liver transplantation plus chemotherapy versus 47 patients to chemotherapy alone. The 5-year overall survival was 57% in the liver transplant arm vs 13% in the chemotherapy alone arm (HR 0.37, 95%CI 0.21-0.65). Among patients undergoing transplantation, a recurrence in the lung, the liver, or other sites subsequently developed in 28, with 15 patients (40%) remaining disease free. These results with transplantation bring into focus the wisdom of offering organ transplants to patients with cancer, an intervention that, while both difficult and expensive, as shown in this trial, can bring some patients a benefit of magnitude much greater than the many expensive therapeutics that barely prolong life and achieve regulatory approvals. The results also highlight another interesting feature of colorectal cancer—that transplant-related immunosuppression does not induce rapid emergence of other disease. This may be consistent with studies showing that colorectal cancer is generally not responsive to immunotherapy.

Together, these 3 abstracts highlight the uniqueness of colorectal cancer, a malignancy unlike most others for which directing treatment toward metastatic sites is not valuable because additional metastatic sites rapidly emerge.

## Treating esophageal cancer

- For resectable locally advanced gastroesophageal junction (GEJ) adenocarcinoma, the optimal therapy remains to be defined with two approaches recommended by guidelines and considered equivalent as regards improvement of overall survival. The two approaches were compared in the ESOPEC trial, a multicenter study in Germany (**Abstract LBA1**)^17^ that compared perioperative FLOT chemotherapy to neoadjuvant chemoradiotherapy (CROSS). Between February 2016 and April 2020, this study randomized 438 patients from 25 sites in Germany with clinical T1N+ or T2-4aNX nonmetastatic resectable esophageal adenocarcinoma (EAC) to receive either perioperative FLOT (5FU/leucovorin/oxaliplatin/docetaxel) or neoadjuvant CROSS (41.4 cGy plus carboplatin/paclitaxel) followed by surgery. The rates of R0 resection were similar in both groups. However, after a median follow-up of 55 months, the median overall survival for FLOT was 66 months and for CROSS 37 months (HR 0.70, 95%CI 0.53-0.92). As with the durvalumab example above, a very large difference in median OS (29 months) achieved a HR of only 0.7, an outcome seen when arms track closely above and below the 50% threshold and cross the 50% boundary many months apart. This observation underscores the importance of looking beyond the numbers at the data to ensure that the true magnitude of benefit is discerned before thinking about and discussing alternatives with patients. In this case, 3-year OS rates of 57.4% and 50.7% were reported, respectively, reflecting a more modest difference between the 2 regimens. Finally, the discussant noted that results from CheckMate-577 support the option of CROSS, then surgery, then adjuvant nivolumab as a viable, well-tolerated alternative.^18^

## Hematologic malignancies

- Abstract LBA6500^19^ reported on ASC4FIRST a randomized clinical trial conducted in multiple global centers that compared asciminib, a novel TKI, to investigator-selected TKIs in patients with newly diagnosed chronic myeloid leukemia (CML). Asciminib is notable for inhibiting kinase activity by binding the ABL myristoyl pocket of the ABL kinase rather than the ATP-binding site targeted by all other TKIs used in the therapy of CML. The median follow-up was approximately 16 months with 201 patients randomized to asciminib and 204 patients to TKIs in current use, including imatinib and all second generation TKIs. The major molecular response (MMR) rate at 48 weeks was 67.7% for asciminib versus 49.0% for all investigator-selected TKIs, *P* < .001, but importantly was not significant when comparing only the half of patients treated with a second generation TKI, a comparison that included patients with a median age of 43 years compared to patients aged 54.5-56 years in the imatinib comparison. In a disease with a median age of 66, a number 10-20 years older than that of study participants, whether efficacy against imatinib but not the second-generation TKIs resulted from a less potent imatinib challenger or was discernible only in older patients, whose rate of death from CML is much higher, remains to be resolved. If felt to be due to the age of the patients, dose adherence will be important given the large imbalance in treatment discontinuation in the older patients—14.9% of those randomized to asciminib compared to 36.3% of imatinib recipients. The latter may be explained in part by the better tolerability of asciminib with fewer grade ≥3 adverse effects, a not surprising observation given the broad similarity of ATP-binding pockets targeted by established TKIs that can lead to more off-target effects. Asciminib may become the new standard of care for CML.^20^
- The German Hodgkin Study Group presented further follow up of their randomized trial in patients with advanced Hodgkin lymphoma (Abstract LBA7000).^21^ The study included 1482 patients with advanced Hodgkin lymphoma who were randomized to dose intense regimens—either BrECADD (brentuximab vedotin, etoposide, cyclophosphamide, doxorubicin, dacarbazine, dexamethasone) or BEACOPP (bleomycin, etoposide, doxorubicin, cyclophosphamide, vincristine, procarbazine, prednisone). With a median follow-up of 4 years, the 4-year PFS was 94.3% for BrECADD vs 90.9% for BEACOPP (HR 0.66, 95%CI 0.45-0.97). This demonstrated the value of does-intense regimens in advanced Hodgkin lymphoma, and the incremental benefit from brentuximab vedotin in combination with chemotherapy, even as nivolumab combined with AVD chemotherapy, presented at ASCO 2023, generated a better 12-month PFS of 94% compared to brentuximab vedotin with AVD, of 86% (LBA4 ASCO 2023).^22^

## Palliative care in oncology

- A groundbreaking study published by Temel et al. (2010) demonstrated that the provision of early palliative consultation to patients with stage IV lung cancer led to significant improvements in quality of life,^23^ and this approach has become incorporated into national guidelines. Nonetheless, the provision of early palliative care has not been widely adopted. At ASCO 2024, Temel et al. presented 2 studies to increase implementation. At the plenary session, a randomized trial was presented in which 1250 patients with advanced NSCLC within 12 wk of diagnosis were recruited over 5 years at 22 cancer centers (Abstract LBA3).^24^ They were randomized to meet with a palliative care clinician every 4 wk by telehealth or in person. At 24 wk, the quality-of-life scores were equivalent in the 2 groups, suggesting that this approach could be effectively utilized. In a second randomized trial, Temel enrolled 507 patients with advanced NSCLC between February 2018 and December 2022 at 3 cancer centers (Abstract 12000).^25^ In this trial, patients were randomized to a simplified, stepped palliative care (SPC) vs the usual palliative care visit every 4 wk. Patients in the SPC arm, after the initial visit, met with the palliative care physician only at the time of a change in cancer treatment or after a hospitalization. At 24 wk, the mean number of physician visits was lower for the SPC arm than the usual care arm (2.44 vs 4.70, *P* < .0001). In addition, the quality-of-life scores were noninferior for the intervention arm.^26^ These two studies—one employing telehealth, and the other a stepped approach—provide lesser resource intense palliative care approaches that could allow for its effective delivery to a larger fraction of patients.

## Prevention of HPV-related malignancies

- Human papillomavirus (HPV) is responsible for a variety of squamous cell malignancies in both men and women, including cervical cancer, cancers of the anogenital area (vulva, vagina, penis, anus), and HPV-related cancers of the head and neck. The development of an effective vaccine for these viruses led in the United States to the recommendation starting in 2006 of administering the vaccine to girls ages 11-12 and starting in 2010 to variable recommendations for males.^27^ Since these cancers primarily occur in middle and older age populations, one will only get an indication of the effectiveness of the vaccine once the population reaches middle age. A retrospective cohort study was performed using the TriNetX United States Collaborative Network, which compiles data from healthcare organizations for clinical trials and health outcomes research purposes (Abstract 10507).^28^ The investigators identified patients 9-39 years of age who underwent HPV vaccination at least 5 years earlier (male *n* = 760 540; female *n* = 945 999) and those with no HPV vaccination. They found that males had a reduced risk of head and neck cancer (odds ratio (OR) 0.44, 95%CI 0.26-0.73). Females had a decreased risk of cervical cancer (OR 0.71, 0.52-0.96) but did not have significantly decreased risk of head and neck or vulvar/vaginal cancer. These are very early signs of the vaccine’s effectiveness, but the numbers still remain small for certain of the malignancies.

## Conclusions

In sum, the abstracts we have presented are some of those we found most thought-provoking. As a whole many are forward looking but not paradigm-shifting, showing that new agents still require significant work to find the best way to incorporate them into the armamentarium.
